# Bystander T-Cells Support Clonal T-Cell Activation by Controlling the Release of Dendritic Cell-Derived Immune-Stimulatory Extracellular Vesicles

**DOI:** 10.3389/fimmu.2019.00448

**Published:** 2019-03-12

**Authors:** Marthe F. S. Lindenbergh, Daniëlle G. J. Koerhuis, Ellen G. F. Borg, Esther M. van ‘t Veld, Tom A. P. Driedonks, Richard Wubbolts, Willem Stoorvogel, Marianne Boes

**Affiliations:** ^1^Department Biochemistry and Cell Biology, Faculty of Veterinary Medicine, Utrecht University, Utrecht, Netherlands; ^2^Department of Pediatrics and Laboratory of Translational Immunology, University Medical Center Utrecht, University Utrecht, Utrecht, Netherlands

**Keywords:** dendritic cells, T-cells, extracellular vesicles/exosomes, HLA class I, antigen presentation

## Abstract

Extracellular vesicles (EV) that are released by immune cells are studied intensively for their functions in immune regulation and are scrutinized for their potential in human immunotherapy, for example against cancer. In our search for signals that stimulate the release of functional EV by dendritic cells we observed that LPS-activated human monocyte-derived dendritic cells (moDC) changed their morphological characteristics upon contact with non-cognate activated bystander T-cells, while non-activated bystander T-cells had no effect. Exposure to activated bystander T-cells also stimulated the release of EV-associated proteins by moDC, particularly CD63, and ICAM-1, although the extent of stimulation varied between individual donors. Stimulation of moDC with activated bystander T-cells also increased the release of EV-associated miR155, which is a known central modulator of T-cell responses. Functionally, we observed that EV from moDC that were licensed by activated bystander T-cells exhibited a capacity for antigen-specific T-cell activation. Taken together, these results suggest that non-cognatei interactions between DC and bystander T-cells modulates third party antigen-specific T-cell responses via EV.

## Introduction

Dendritic cells (DC) are sentinel cells that survey the microenvironment of barrier tissues such as the skin or mucosae for the presence of danger-associated molecules and pathogens. DC initiate adaptive immune responses by presenting peptides from exogenously acquired antigens onto Major Histocompatibility Complex (MHC) class I molecules to CD8+ T-cells (cross-presentation), or onto MHC class II molecules to CD4+ T-cells (1). Upon sensing pathogen-derived molecules, such as lipopolysaccharide (LPS), DC mature into potent antigen presenting cells (APCs) that mobilize toward lymphoid tissues where they can encounter and stimulate rare antigen-specific naive B- and T-cells. When antigen-specific CD4+ T-cells are stimulated by cognate MHC class II-peptide complexes on activated DC, they increase their CD40 ligand (CD40L) expression, which in return can license DC to mature further into an APC phenotype that can prime the development of antigen-specific CTL (2, 3). Activated CD4+ T-cells can, however, also induce costimulatory DC in the absence of any innate DC priming, affecting all DC in the microenvironment, including those that lack specific antigen (4). Similarly, CD8+ T-cells can also induce DC maturation independently of CD40, in absence of innate stimuli (5, 6). Thus, DC phenotype and efficiency is shaped by the combined actions of innate and adaptive signals. It is not well understood how exactly bystander T-cells improve the efficiency with which DC activate cognate T-cells. To further elucidate this process, we set out to investigate the effect of bystander T-cells on the release of extracellular vesicles (EV) by DC. DC secrete antigen presenting EV which, depending on the status of the originating DC, can have either tolerogenic or immune-stimulatory effects when interacting with T-cells (7, 8). The capacity of EV to present antigen depends on the maturation status of the parental DC (9), and the presence of costimulatory molecules on the EV (10). We report here that incubation with activated bystander T-cells changed the phenotype of LPS-experienced DC, and modulated their EV-release. Functionally, EV that were released by DC upon contact with activated bystander T-cells elicited stronger peptide-specific T-cell responses than did EV induced by non-activated bystander T-cells. We propose a role for DC-derived EV in inflammatory conditions, where interactions between matured DC and activated bystander T-cells stimulate the release of EV by DC, to support subsequent antigen-specific T-cell activation.

## Materials and Methods

### Cell Culture and Processing

Peripheral blood mononuclear cells (PBMCs) from healthy donors were isolated from lithium heparinized blood samples using Ficoll isopaque density gradient centrifugation (GE Healthcare). CD14+ monocytes were enriched using positive selection with CD14+ MicroBeads (Miltenyi Biotec). CD14+ cells were cultured in 6-wells plates (Nunclon, Thermo Scientific) at a concentration of 1 to 1.5 × 10^6^/mL at 37°C and 5% CO_2_ for 5 days in RPMI 1640 GlutaMAX (Gibco), 1% Penicillin/Streptomycin (Gibco) and 20% heat inactivated, 0,2 μM filtered FCS (Biowest) supplemented with 450 U/mL rhGM-CSF (Immunotools) and 300 U/mL IL-4 (Immunotools). Cytokines were replenished after 3 days. Purity of CD14+ cells was determined using flow cytometry, and only cultures containing ≥90% CD14+ cells were used.

T-cells were isolated from the CD14-depleted fraction of PBMCs (from healthy donors) by negative selection using the Pan T Cell Isolation kit (Miltenyi Biotec). Isolated T-cells were cultured in RPMI 1640 (Gibco) containing 1% Penicillin/Streptomycin (Gibco), 1% L-glutamin (Gibco) and 10% heat inactivated, 0.2 μM filtered FCS (Biowest). T-cells were activated with 5 ng/mL phorbol 12-myristate 13-acetate (PMA) (Sigma) and 1 μg/mL Ionomycin (Sigma), or alternatively, when indicated, by Dynabeads coated with anti-CD3 and anti-CD28 (Gibco). After 16 h, cells were harvested and, if applied, magnetic Dynabeads were removed. Non-activated and activated T-cells were mildly fixed with 0.4% paraformaldehyde (PFA) (Electron Microscopy Sciences) in PBS for 30 s. The fixative was quenched with 0.2M glycine, and T-cells were washed by centrifugation, twice with cold PBS (Gibco) and once with culture medium. After washing, T-cells were resuspended in EV-free moDC medium and stored at 4°C. EV-free medium was prepared by ultracentrifugation for 18 h (100,000 × g at 4°C). When indicated, moDC were activated by culturing for 4 h in the presence of 100 ng/mL ultrapure LPS (*E. coli* strain O111:B4, Invivogen). Activated moDC were washed by centrifugation to remove LPS and resuspended in EV-free medium. Subsequently, fixed T-cells were added 1:1 to the activated moDC. For microscopy purposes, T-cells were co-cultured with moDC either in direct contact or in separation by transwell inserts with a pore size of 1.0 μm (Greiner bio-one). After 18 h, ice cold PBS was added in excess, and cells and culture supernatant were harvested. Blood from healthy volunteers was obtained following institutional ethical approval (www.umcutrecht.nl/METC), METC protocol number 07-125/C. The experiments abide by the Declaration of Helsinki principles for human research ethics.

### EV Isolation, Protein Deglycosylation, and Western Blotting Analysis

EV were collected from culture media by differential (ultra)centrifugation at 4°C, as published (11). Briefly: cells were removed by centrifugation twice for 10 min at 200 × g, followed by two times 10 min at 500 × g at 4°C. Next, the samples were centrifuged sequentially at 10,000 × g (30 min, 8,900 rpm, 4°C) and at 100,000 × g (65 min, 28,000 rpm, 4°C) in polyallomer tubes (Beckman Coulter) using a swing-out rotor (SW-40, Beckman Coulter). For antigen presentation assays, 100,000 × g pellets were resuspended in EV-free culture medium and stored at 4°C. For Western blotting analysis, 100,000 x g pellets were lysed in non-reducing SDS-PAGE sample buffer.

For deglycosylation assays, cell suspensions were lysed in Triton X-100 buffer with complete protease inhibitor mix (Roche). Subsequently, the lysate was spun at 12,000 rpm, and the supernatant was heated to 100°C for 10 min followed by overnight deglycosylation at 37°C using either EndoH or PNGase F (New England Biolabs) in presence of their respective glycoprotein buffers. After deglycosylation, 4x SDS-PAGE sample buffer was added to the samples.

For Western blotting, proteins were separated by 10% SDS-PAGE and transferred to 0.45 μm polyvinylidene difluoride (PVDF) membrane (Merck Millipore). The blots were blocked and incubated with antibodies in PBS containing 0.2% gelatin from cold water fish (Sigma) and 0.1% Tween-20. Immunodetection was performed using mouse anti-human CD9 (clone HI9a; 1:2,000; Biolegend), mouse anti-human CD63 (clone TS63; 1:2,000; Abcam), mouse anti-human CD81 (clone B-11; 1:400; Santa Cruz), or mouse anti-human HLA-B,C (some A) (clone HC-10; 1:400; kindly provided by E.J.H.J. Wiertz), followed by HRP-conjugated goat anti-mouse IgG and IgM (1:10,000; Jackson). HRP activity was detected using ECL (SuperSignal West Dura Extended Duration Substrate, Thermo Scientific) and a ChemiDoc MP Imaging System (BioRad). Relative intensity data were analyzed using Image Lab V5.1 (BioRad).

### Microscopy

For differential interference microscopy (DIC), moDC and T-cells were mixed at concentrations of 1.25 × 10^5^ cells/mL each and co-cultured overnight as indicated above. Imaging was performed using a Leica DM IRBE microscope with LMC40 and 40x objective combined with a Leica D-LUX 3 (LMS) camera. For confocal microscopy, moDC and T-cells were seeded on glass coverslips, each at 2 × 10^5^ cells/mL. After overnight incubation, cells were fixed for 30 min with 4% paraformaldehyde in 0.1 M Phosphate buffer at pH 7.4, followed by quenching and permeabilization in PBS containing 20 mM NH_4_Cl, 2% BSA (Sigma) and 0.1% w/v saponin (Sigma). Subsequent labeling and washing was performed in PBS containing 2% BSA and 0.1% saponin. HLA class II was labeled with CR3/43 (1 μg/mL, DAKO) for 45 min, followed by Alexa-488 labeled goat anti-mouse IgG (1 μg/mL, Invitrogen) for 30 min. Nuclei were labeled with DAPI (4′,6-diamidino-2-phenylindole, 23.8 μM, Thermo Fisher Scientific) for 1 min. Labeled coverslips were finally washed with water and embedded in Prolong Diamond (Thermo Fisher Scientific). Images were acquired using a NIKON A1R confocal microscope with 40x Plan Apo objective (NA 1.3), with regular lasers and filter settings to detect DAPI and Alexa488. Overviews of the cultures were generated by scanning 7 × 7 image fields at 3 positions in the Z axis at 1.5 μm steps. Representative regions of 300 × 300 pixels were selected and processed in NIS elements 5.02 (Nikon Microsystems, Europe). Fluorescence images were captured with identical settings, and maximum intensity projection was performed.

### RNA Isolation and qPCR

Small RNA was isolated from EV pellets using the miRNeasy micro kit according to manufacturer's instructions (Qiagen). The RNA yield and size profile were analyzed using the Agilent 2100 Bioanalyzer with Pico 6000 RNA chips (Agilent Technologies). cDNA was generated from 3 μL EV-derived small RNA using the miScript RT2 kit (Qiagen). At least 20 pg RNA was used per qPCR reaction, and mixed in 8 μL reactions in triplicate with 100 nM primers (IDT, see Table 1) and 4 μL SYBR Green Sensimix (Bioline). Control samples without RT-enzyme were used to confirm absence of genomic DNA and non-specific amplification. Cycling conditions were 95°C for 10 min, followed by 50 cycles of 95°C for 10 s, 57°C for 30 s, and 72°C for 20 s. All PCR reactions were performed using the Bio-Rad iQ5 Multicolor Real-Time PCR Detection System. Quantification cycle (Cq) values were determined using Bio-Rad CFX software with automatic baseline settings, averages of triplicates were used for further analysis. Thresholds were set in the linear phase of the amplification curve. Forward primer sequences for miRNA targets were designed based on sequence information from miRBase, the U6 primer sequence was taken from Galiveti et al. (12) (Table 1). As a reverse primer, the miScript universal reverse primer was used in all reactions.

**Table 1:** Forward primer sequences.

**RNA species**	**Primer sequence from 5^**′**^ to 3^**′**^**
hsa-miR-30b-5p	TGTAAACATCCTACACTCAGCT
hsa-miR-155-5p	TTAATGCTAATCGTGATAGGGGT
hsa-miR-146a-5p	TGAGAACTGAATTCCATGGGT
U6-F	CTCGCTTCGGCAGCACA

### Preparation of NLV-Expanded T-Cells and NLV Specific T-Cell Activation Assay

For antigen-specific T-cell activation experiments we exclusively used moDC and bystander T-cells from HLA-A^*^02.01 positive donors, as determined by flow cytometry. CD8 T-cells recognizing the HCMV (human cytomegalovirus) pp65-derived peptide (NLV) were isolated and expanded as described (13) with minor modifications. In brief, T-cells from an HLA-A^*^A02.01 positive donor were cultured with THP-1 cells presenting NLVPMVATV peptide (Proimmune). After 1 week, T-cells positive for HLA-A2/pp65_495−503_ tetramers were sorted and incubated with irradiated, T-cell depleted (using positive CD3+ sort from Miltenyi Biotec), PBMCs (1 × 10^5^ cells/mL, irradiated with 70 Gy) from three healthy donors. The cells were cultured in the presence of 1 μg/mL leucoagglutinin PHA-L (Sigma-Aldrich) and 120 U/mL of recombinant human IL-2 (Immunotools) and re-stimulated and expanded during several culturing cycles before freezing in aliquots. Aliquots of expanded T-cells were thawed for each experiment, and incubated in medium containing 0.1% Monensin/Golgistop (BD). Portions of the EV isolates were pre-incubated for 1 h with 10^−7^ M NLVPMVATV peptide (NLV) prior to their addition to the T-cells. EV isolated from 200.000 moDC were added to 50.000 T-cells in a total volume of 50 μL in a round-bottom 96-wells plate (Thermo Scientific), and incubated for 4 to 5 h before assaying by flow cytometry intracellular cytokines and cell surface marker expression.

### Flow Cytometry

Cells were blocked in flow cytometry buffer (PBS with 0.5% BSA and 0.02% NaN_3_) supplemented with 0.5% NMS (Fitzgerald 88R-M002). For intracellular staining, cells were permeabilized using cytofix/perm buffer (BD) and washed in perm/wash buffer (BD). CD3 was detected using Pacific Blue (PB)-labeled mouse-anti human CD3 (clone UCHT-1; 1:50; Beckman Coulter), CD8 with Fluorescein (FITC)-labeled mouse anti-human CD8 (clone RPA-T8; 1:25; BD), CD107a (LAMP-1) with Phycoerithrin (PE)-labeled mouse anti-human CD107a (clone H4A3; 1:25; BD), IFNγ with PE-Cy7-labeled mouse anti-human IFNγ (clone 4S.B3; 1:200; BD), TNFα with Allophycocyanin (APC)-labeled mouse anti-human TNFα (clone mab11; 1:200; Sony Biotech), HLA-A2 with PE-labeled mouse anti-human HLA-A2 (clone BB7.2; 1:100; Biolegend), and CD69 with FITC-labeled mouse anti-human CD69 (clone FN50; 1:50; Miltenyi Biotec). PE-labeled murine IgG1,k (clone MOPC-21; BD) and FITC-labeled murine IgG1,k (clone MOPC-21; BD) were used as isotype controls. Data were collected on a FacsCanto II (BD) flow cytometer and analyzed with FlowJo 10.0 software (Treestar).

### Statistical Analysis

Significance of differences was determined by two-sided paired *t*-tests using GraphPadPrism 7 software. When indicated, we used a Wilcoxon's signed rank test to demonstrate consistent differences between paired observations. *P* < 0.05 were considered statistically significant.

## Results

### Response of Activated moDC to Activated Bystander T-Cells

**Figure 1:**
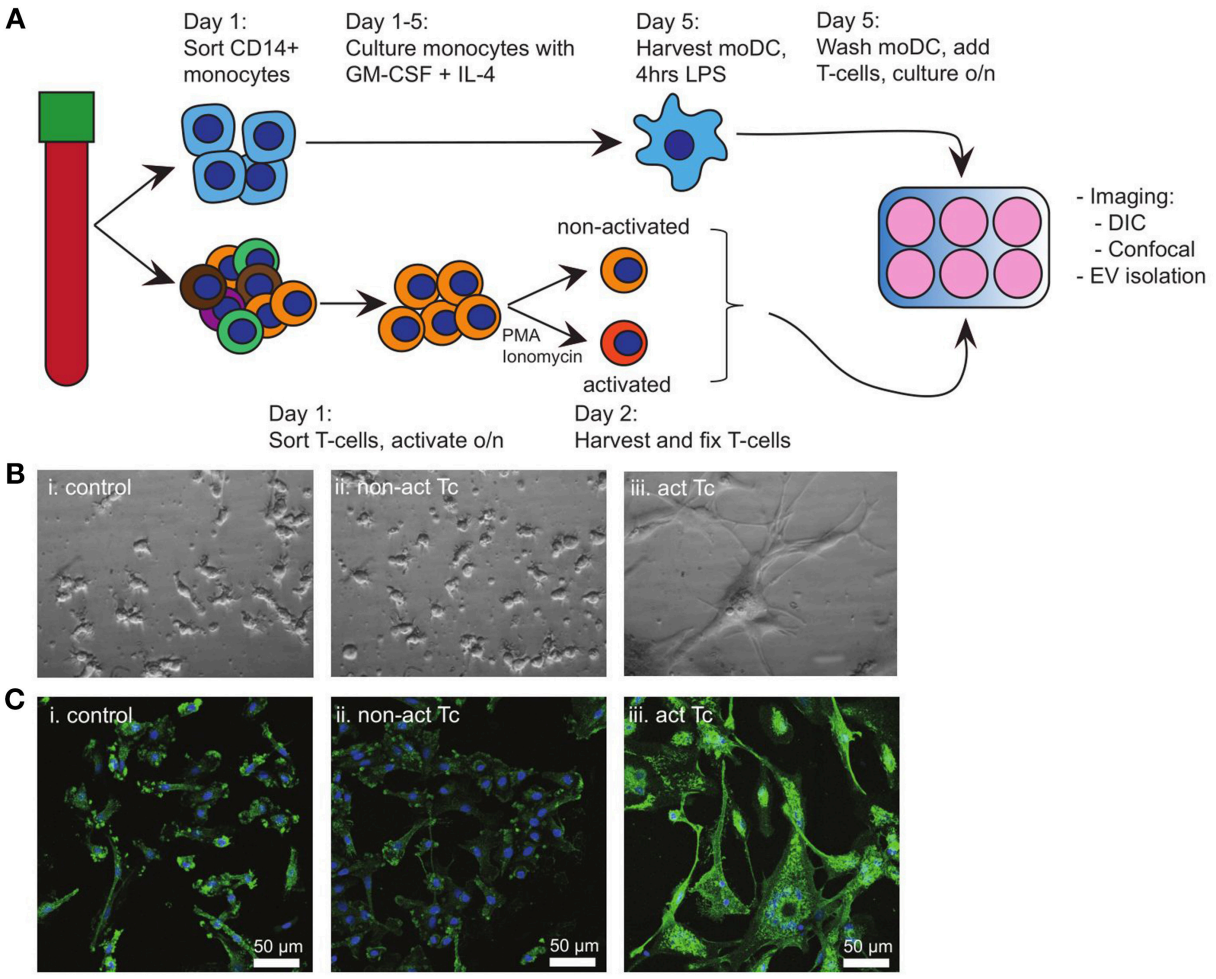
Activated T-cells alter the phenotype of LPS-experienced moDC. **(A)** Schematic overview of the experimental design. Monocytes and T-cells were sorted separately from the same PBMC-isolate. A proportion of the T-cells (bystander T-cells) was activated o/n with PMA and Ionomycin, after which non-activated and activated T-cells were mildly fixed in 0.4% PFA. Meanwhile, moDC were differentiated from monocytes during 5 days culturing in the presence of GM-CSF and IL-4, and subsequently activated for 4 h with LPS. Subsequently, LPS-experienced moDC and bystander T-cells were incubated o/n in a 1:1 ratio. **(B)** DIC images of o/n cultured moDC. (i) control (LPS-matured only); (ii) no effect of fixed, non-activated T-cells; (iii) stretching and clustering of moDC in the presence of fixed, activated bystander T-cells. Representative images from one out of three independent experiments are shown. All images were acquired using the same magnification. **(C)** Fluorescence microscopy detection of HLA class II on moDC (green), and nuclei (blue) in samples similar to **(B)**. Representative images from one out of three independent experiments are shown; (i) control (LPS-matured only); (ii) no effect of non-activated T-cells on moDC phenotype; (iii) moDC stretch, cluster, and HLA II expression is increased upon incubation with activated T-cells. HLA class II expression in increased. Bars indicate 50μm.

Breaching of epithelia that cover barrier tissues elicits antigen-specific immune activation, which relies on the traffic of maturing tissue-derived DC to deliver antigen-specific cues to secondary lymphatic organs. Here, such DC interact most frequently with non-cognate bystander T-cells, some of which might provide further maturation signals to the DC. In our search for signals from bystander T-cells to maturing DC, we first examined the effects of bystander T-cells on LPS activated autologous moDC (Figure 1A). To eliminate any possible contribution of EV from bystander T-cells, non-activated or T-cells that had been activated with PMA and Ionomycin were mildly fixed with PFA prior to addition to moDC (14). Fixation did not change the exposed activation markers on the T-cells (Supplementary Figure 1A). Addition of LPS alone to moDC (control) resulted in the outgrowth of dendrites, characteristic for DC maturation (Figure 1Bi). Subsequent exposure to non-activated bystander T-cells did not induce further morphological features of maturation (Figure 1Bii). In contrast, exposure to activated bystander T-cells triggered LPS-experienced moDC to form large clusters of stretched cells, which firmly adhered to the culture dish (Figure 1Biii). This effect was not caused by the fixation of the bystander T-cells, as moDC incubated with bystander T-cells that had not been chemically fixed showed the same characteristics (Supplementary Figure 1B). Furthermore, the same DC phenotype was observed when the DC were incubated with T-cells that were activated with anti-CD3/CD28 coated Dynabeads, indicating independence of the method applied for T-cell activation (Supplementary Figure 1Ci,ii). We were unable to dissociate individual moDC from these induced clusters of cells, precluding flow cytometric analyses (our unpublished observations). Instead, we resorted to confocal microscopy to visualize the expression and distribution of human leukocyte antigen (HLA) class II complexes. Non-activated bystander T-cells did not modulate the expression or distribution of HLA class II in already matured LPS-experienced moDC (Figure 1Ci,ii). Activated bystander cells, however, upregulated HLA class II expression (visualized as an increased intensity of fluorescence), both intracellularly and at the plasma membrane (Figure 1Ciii). When activated T-cells were separated from the DC by using a transwell insert expression of HLA II was not increased (Supplementary Figure 1Ciii compared to Supplementary Figure 1Cii), indicating a requirement for direct contact of DC with activated T-cells. In conclusion, interactions with autologous activated bystander T-cells resulted in phenotypic maturation of moDC, whereas encounters with non-activated bystander cells did not.

### Activated Bystander T-Cells Induce Release From moDC of EV-Marker Proteins and miRNA Species

**Figure 2:**
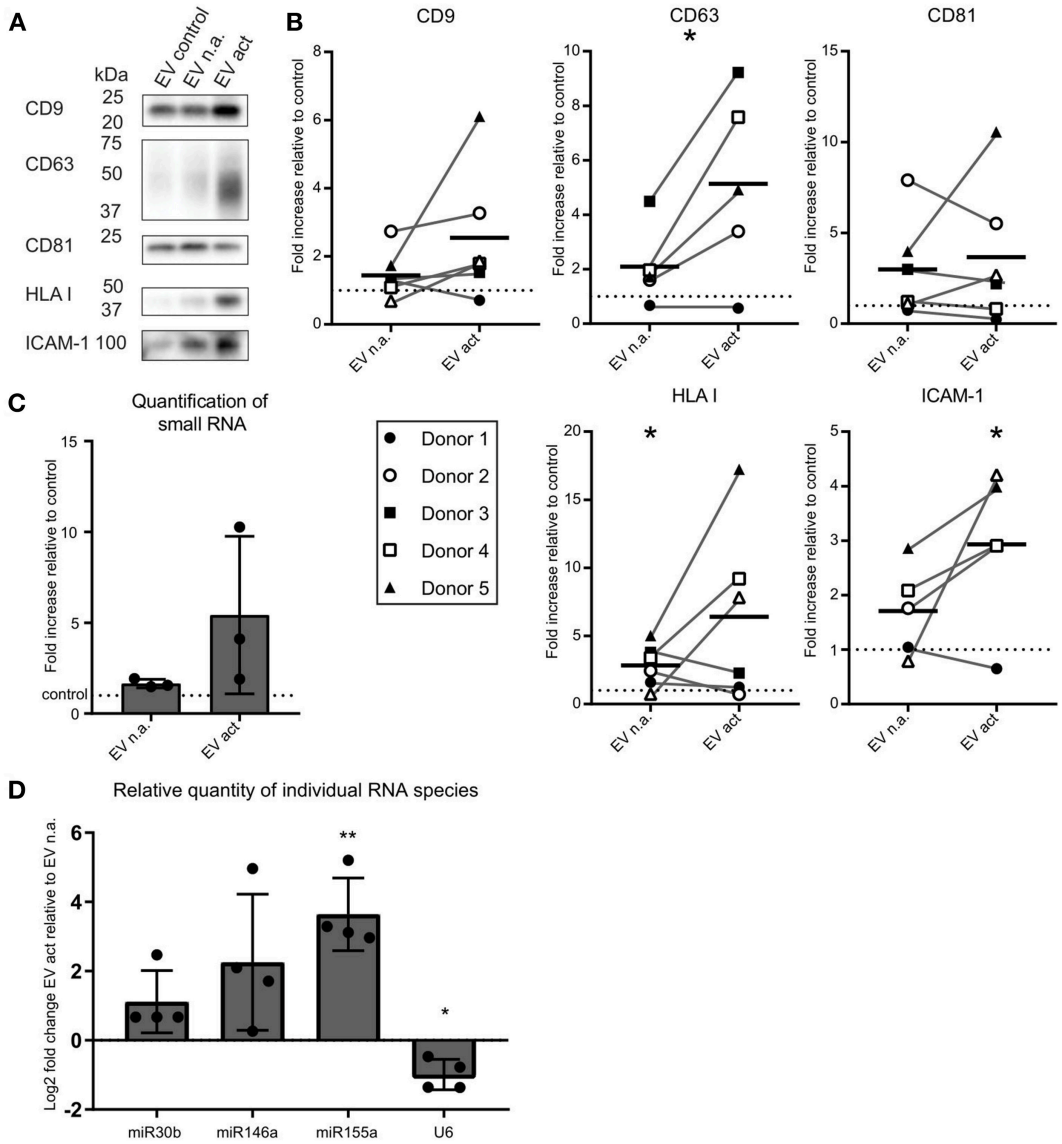
Activated bystander T-cells modulate the release of EV-markers by LPS-stimulated moDC. **(A)** Western blot detection of markers in EV collected from moDC incubated with LPS only (control), or additionally with non-activated (n.a.) or activated (act) bystander T-cells. EV were isolated from the culture supernatant of equal numbers of moDC per condition. Representative experiment out of six independent experiments is shown. **(B)** Quantification of data as in **(A)** for five or six independent donors from six independent experiments. Independent experiments (donors) are each represented by individual symbols. Values are plotted relative to the signal obtained for the control condition ( moDC incubated with LPS only), represented by the dotted line. Horizontal lines indicate the mean. Asterisks placed above symbols indicate a significant difference between the data represented by these symbols and the control condition (Paired *t*-test, *p* < 0.05). Asterisks placed between the columns with symbols indicate a significant difference between the data represented by the two conditions (Paired *t*-test, *p* < 0.05) **(C)** Release of total small RNA in EV derived from LPS-experienced moDC is increased upon addition of bystander T-cells, especially activated bystander T-cells. Quantification of relative amounts of small RNA isolated from EV pellets from co-culture supernatant, as measured with Bioanalyzer Pico. Data are from three independent donors in three independent experiments. The control condition is represented by the dotted line. **(D)** qPCR detection of specific small RNA species in EV pellets collected from LPS-matured moDC incubated with non-activated (EV n.a.) or activated (EV act) bystander T-cells. Changes in (mi)RNA levels of EV act are depicted as log2-fold-change relative to EV n.a. U6 refers to the small nuclear snRNA species U6. Quantification of data from four independent donors in four independent experiments. Asterisks placed above columns indicate a significant difference between the data represented by the column and the control condition. Paired *t*-test *p < 0.05, **p < 0.01.

We next asked whether activated bystander T-cells influenced the release of EV by moDC. As before, to ensure that we collected moDC-derived EV only, we chemically fixed the bystander T-cells prior to addition to moDC. After overnight co-incubation of moDC and fixed bystander T-cells, EV were collected from culture media by differential (ultra)centrifugation. With this procedure, large EV and cell debris were discarded by centrifugation up to 10,000 × g, and only small EV were collected during a final centrifugation step at 100,000 × g (11). These EV were analyzed by Western blotting for the presence of EV marker tetraspanins (CD9, CD63, CD81), the antigen-presenting molecule HLA class I, and intercellular adhesion molecule ICAM-1 (Figure 2A). From here on we refer to EV released by moDC upon contact with non-activated bystander T-cells as “EV n.a.” Their counterparts, released by moDC upon contact with activated bystander T-cells, are referred to as “EV act.” We found that the release of EV-associated markers by moDC remained largely unaffected by the presence of non-activated bystander T-cells (EV n.a.). In contrast, activated bystander T-cells stimulated the release of EV-associated proteins by moDC (EV act). Due to variation between donors, however, significant increases were established only for HLA-I (1.8-fold, ±1.6, *n* = 6, *p* = 0.0344), CD63 (3.0-fold, ±2.3, *n* = 5, *p* = 0.0407), and ICAM-1 (1.9-fold, ±1.4, *n* = 5, *p* = 0.0375) for the combined results from five to six independent donors (Figure 2B). Interestingly, incubation with activated bystander T-cells resulted in a slight decrease in the apparent molecular weight of CD63 (Figure 2A). Of note, this change in migration by SDS PAGE was not exclusive for the EV-associated CD63, as we also observed this for CD63 in the cell lysate of moDC (Supplementary Figure 2). Further analysis suggests that the change in mobility is caused by differences in glycosylation of the protein (Supplementary Figure 2).

We next asked whether encounters with bystander T-cells would influence the loading of mi-RNA-species into moDC-derived EV. We first analyzed the relative quantity of total small RNA isolated from EV from equal numbers of moDC at different experimental conditions. Relative to the LPS only condition, we observed a significant, albeit small, increase in total small RNA in response to EV n.a. (*p* = 0.0419) (Figure 2C). The increase in release of total EV associated small RNA by moDC in response to interaction with activated bystander T-cells was highly variable between donors and did not reach significance (Figure 2C). Next, the abundance of specific miRNA species with known immune-modulatory effects in moDC-derived EV was determined by qPCR. Because of the current lack of generally applicable reference genes to normalize RNA abundance in EV from different conditions (15), we quantified changes in specific miRNAs in EV isolated from equal numbers of moDC (Figure 2D). We found that miR-155 was increased by a Log2-fold change of 3.6 (±1.0, *n* = 4, *p* = 0.061) in EV act compared to EV n.a. Additionally, we observed an increase in miR-146a and miR-30b in EV act for some of the donors, although the difference between EV act and EV n.a. did not reach significance for these miRNA species. U6, which is often used as a reference gene in EV (16), did not follow the trends observed for the different miRNAs. In conclusion, activated bystander T-cells increased the release of specific small RNAs in moDC-derived EV, such as the pro-inflammatory miR-155, which may have an effect on the immunogenic potential of these EV.

### Encounter With Activated Bystander T-Cells Drives Secretion of Immune-Stimulatory EV by DC

**Figure 3:**
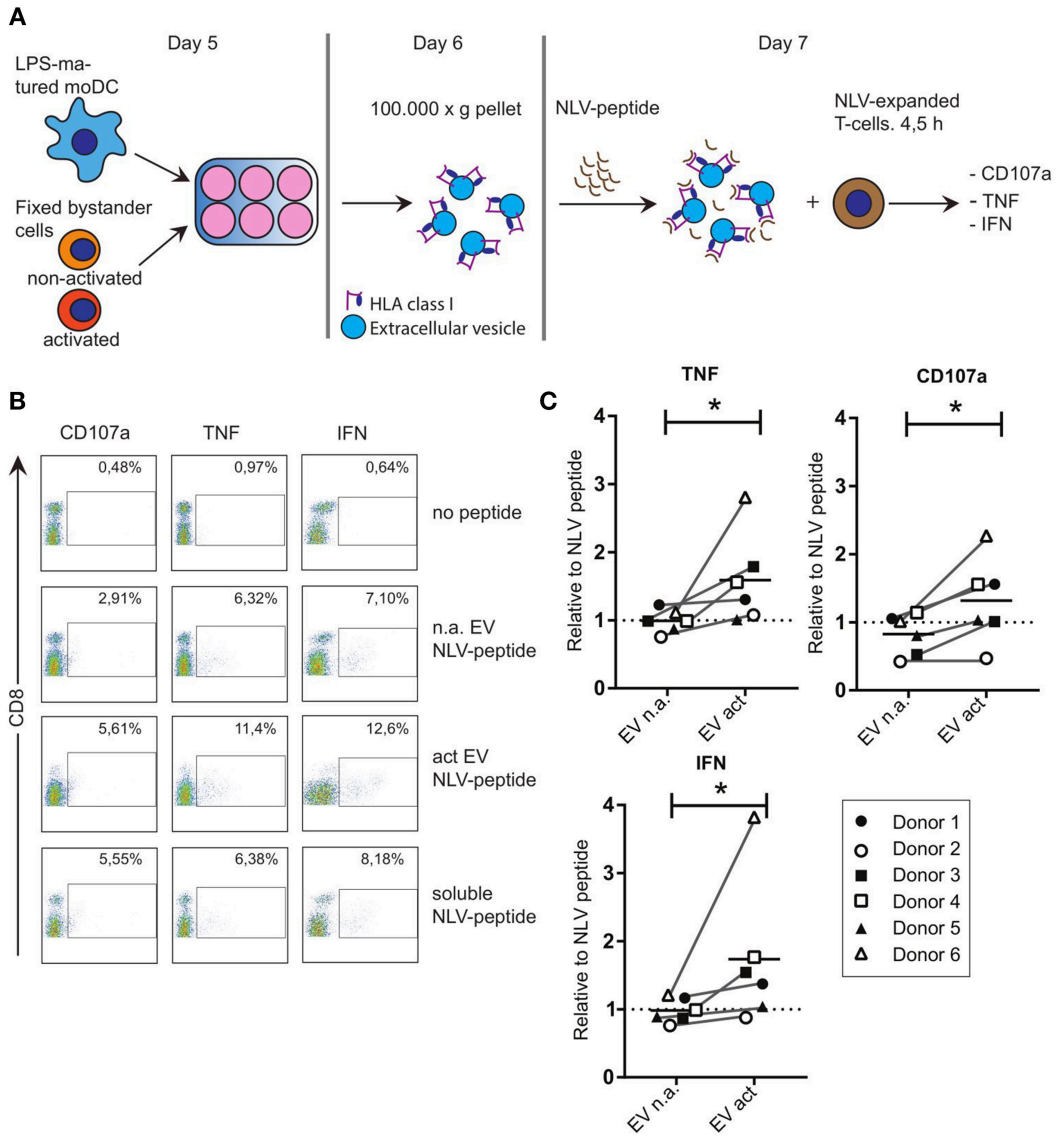
EV that are released from moDC in response to non-cognate interactions with T-cells can stimulate antigen specific T-cell responses. **(A)** Schematic representation of the experimental workflow. LPS-experienced DC were incubated o/n with fixed, activated or non-activated bystander T-cells. The next day, EV were collected from the culture medium and incubated with NLV-peptide for 1 h. Subsequently, NLV-expanded CD8+ T-cells were added to the EV and incubated for 4.5 h, followed by flow cytometric analysis of cognate activation markers. **(B)** Flow cytometric detection of CD8 and cognate activation markers CD107a, TNF and IFN. CD8+ T-cells were gated (not shown), after which CD8+ T-cells with increased expression of the indicated activation markers (top) were gated (boxes) and quantified as a percentage of the total number of CD8+ T-cells (top right in each plot). Representative experiment out of six independent experiments. **(C)** Quantification of T-cell activation data as in **(B)**, for six independent experiments (each represented by different symbols). NLV-specific T-cell activation by moDC-derived EV induced by non-activated bystander T-cells (EV n.a.) or activated bystander T-cell-induced EV (EV act) are plotted relative to the NLV-only condition (no EV present). Horizontal lines indicate the mean. All donors showed a significant increase in cognate T-cell activation in EV act compared to EV n.a. Wilcoxon's signed rank test *p < 0.05.

Considering the reported immune-modulatory functions of the miRNA species that we found to be increased in EV act, we set out to test the potential of these EV in driving antigen-specific immune responses. Others showed that primed- but not naive T-cells, can be activated by EV directly in the absence of bystander DC (17). To test the ability of moDC-derived EV to induce antigen-specific CD8+ T-cell activation, we resorted to the use of our established system of CD8+ T-cells, which were expanded in presence of the CMV pp65-derived NLV-peptide (13). Of note, only a subpopulation of these T-cells is NLV-reactive after expansion, and these primed cells do not display activation markers until re-stimulated. LPS-experienced moDC were incubated with fixed bystander T-cells and EV isolated from the culture medium as above. Isolated EV were subsequently incubated with the NLV peptide for 1 h to allow passive binding of the high affinity NLV-peptide to HLA-A2 (3) (Figure 3A). Next, peptide-loaded EV were incubated with NLV-expanded T-cells, after which the T-cells were probed by flow cytometry for the presence of activation markers. In absence of the relevant peptide we only saw background labeling of the surface activation marker CD107a, and intracellular activation markers TNF and IFN could not be detected (Figure 3B, top row). Upon incubation with NLV-peptide-loaded n.a. EV, a small proportion of the T-cells was activated (Figure 3B, second row). NLV-loaded EV act consistently stimulated a higher percentage of NLV-specific T-cells to express the three cognate activation markers (Figure 3B, third row). Incubation with NLV-peptide alone, in the absence of EV, also resulted in antigen specific activation, albeit at lower percentages compared to T-cells incubated with NLV-loaded EV act (Figure 3B, last row). This residual activity is likely a result of HLA-A2 expression by and presentation amongst human T-cells themselves. After analyzing six different donors, we found that consistently more T-cells were activated by EV act + NLV as compared to EV n.a. + NLV, as determined by TNF (*p* = 0.0313), CD107a (*p* = 0.0313), and IFN (*p* = 0.0313) (Figure 3C). In conclusion, we demonstrate that moDC increased their release of EV markers in response to interaction with activated bystander T-cells, and that these EV are functionally active in stimulating peptide-specific CD8+ T-cells.

## Discussion

DC in peripheral tissues serve as sentinels of the immune system. When DC encounter pathogens in tissues such as the lungs and intestines, they are triggered to migrate and transport antigens toward secondary lymphoid tissues to prime antigen-specific T-cell responses. During transit to lymphoid tissues, DC undergo staged maturation to support their later role in T-cell activation. Within the lymph node, different cell types influence each other's functionality. In depth knowledge on the specific interactions and their consequences is paramount for the design of effective vaccines and immune therapies. Dendritic cells are especially of interest, since they integrate signals of the innate immune system with the adaptive immune system, ultimately determining their efficiency in activating antigen-specific T-cells (1). Interactions with activated bystander T-cells have been shown to increase the efficiency with which DC activate cognate third party T-cells (4). This is partly mediated via upregulation of the expression of costimulatory molecules and by increased production of the T-cell stimulatory cytokine IL-12 p70 (4). Interestingly, in response to maturation signals, DC also release EV with the capacity to activate cognate T-cells, either directly or via bystander cells (17–20). How and which stimuli most efficiently induce release of DC-derived EV that are potent in cognate T-cell activation remains largely unknown. Therefore, we set out to investigate whether bystander T-cells have a functional effect on the release of DC-derived EV with the capacity to activate cognate T-cells. To this end, we cultured LPS-experienced DC with fixed bystander T-cells, enabling us to isolate EV from DC only, to study their composition and functions. We found that LPS-experienced DC displayed a more activated phenotype upon subsequent incubation with activated bystander T-cells, as established by morphological criteria and MHC class II expression. Moreover, activated T-cells stimulated the release by DC of EV-associated ICAM-1 and CD63 as well as small RNA species, including immune-stimulatory miR-155. Importantly, we found that EV act were significantly more efficient than EV n.a. in activating antigen-specific T-cells. Our data are consistent with earlier observations that DC-derived EV can present antigen and carry costimulatory molecules, and can thus activate primed cognate T-cells directly in the absence of activated bystander DC (10, 17).

EV constitute a heterogeneous group of secreted vesicles that are variable in size and cargo, and different subclasses have been shown to be relevant in cell-to-cell communication for immune modulation (7, 8, 21, 22). The maturation status of the EV-producing DC determines the antigen presentation capacities of these EV (23). EV released by mature DC have the ability to induce cognate T-cell activation alone, or after being transferred to bystander cells (17–20). In a mouse model system, cognate interactions with antigen-specific primed CD4+ T-cells strongly enhanced secretion by DC of EV with the potency to present antigen, while non-cognate DC-T-cell interactions also stimulated EV release by DC, albeit to a lesser extent (24). Our current demonstration that non-cognate interactions between activated human moDC and activated T-cells stimulated the release of antigen-presenting and/or immune-modulating EV by DC is consistent with these previous observations.

The release by moDC of EV-associated HLA I, adhesion molecule ICAM-1 and tetraspanin CD63, as well as miR-155a, was increased in response to activated bystander T-cells. The increase of these markers could be attributed either to an increase of number of EV, changes in protein and miRNA load per individual EV, or changes in composition of different types of EV within the total EV population. Given the limited change in EV marker content, as observed here, it is difficult to discriminate between these possibilities. Indeed, without further purification, precise determination of EV number and characteristics is challenging using currently available techniques (21). As this would require larger quantities of EV than could be isolated with the current setup, we were unable at this stage to more precisely determine dissimilarities between EV released at different experimental conditions. Another complication is that a proportion of the released EV is recruited by activated bystander moDC. Therefore, isolated EV are likely to represent only a subset of the total EV population. Furthermore, in our analysis we omitted large EV that may have been lost in the 10.000 x g pellets. Others found that particularly small EV, pelleting at 100.000 × g, promoted Th1 cytokine secretion (IFN-γ), while large EV pelleting at 10.000 × g stimulated secretion of Th2 cytokines (25). Given that human moDC particularly induce Th1 differentiation (26), we here focused exclusively on small EV, isolated at 100.000 × g and the Th1 cytokines IFN and TNF. Taken together, it should be noted that although our quantitative assessment of EV-associated markers is indicative for either quantitative or qualitative differences in EV release, restrictions of isolation procedures and dynamic processes such as recruitment of (selective) EV populations by bystander moDC and T-cells are likely to have influenced these data.

The presence of ICAM-1 on DC-derived EV, and ICAM-1 dependent recruitment of EV by bystander DC were reported to be essential for their priming activity on naive T-cells (23). However, DC-derived EV can also be recruited by activated T-cells via LFA-1—ICAM-1 interactions (27), and peripheral primed CD8 T-cells could be activated by isolated EV from moDC in the absence of bystander DC (28). Our current demonstration that antigen loaded EV from bystander T-cell stimulated DC could directly activate antigen specific CD8 T-cells is consistent with these earlier observations.

EV contain different RNA molecules (29), that upon transfer can be functional in target-cells (30). EV-associated RNA species are intensively studied for their role in disease (31) and are also scrutinized for their immune-modulatory functions (32, 33). We found an increase in the release of several EV-associated miRNA species by DC in response to activated bystander T-cells. Of the miRNA species tested, particularly miR-155 was highly enriched (approximately 15-fold) under influence of activated bystander T-cells. MiR-155 is known to stimulate both T-cell activation (34) and the differentiation of immune-stimulatory DC (35). Rab27a and Rab27b double-knockout mice are deficient in exosome secretion (36), and found to be refractory to stimulation with LPS, indicating a *in vivo* role for exosomes in the response to endotoxins (37). LPS responsiveness could be rescued by i.p. injection of EV isolated from wildtype DC, but not by EVs from miR-155 knock out DC. These authors also demonstrated that expression of SHIP1 and IRAK-M, which are regulators of LPS responses, are direct targets by EV-associated miR-155 (37). Together with these data, our observations indicate that in EV act-associated miR-155 may be released in response to innate inflammatory conditions (e.g., LPS and abundance of activated T-cells), to support subsequent adaptive immune responses. EV-associated miR-146a-5p also appeared to increase in response to activated bystander T-cells, albeit to a lesser extent. MiR-146a is involved in regulation of Th1 responses by regulatory T-cells (38) and is important to prevent autoimmune responses (39). We found that miR30b-3p was not significantly increased by the presence of bystander T-cells in DC-derived EV. Consistent with this observation, miR30b-3p has been reported to be elevated in tolerogenic DC (40). To be able to compare the relative quantities of miRNAs associated with immune-stimulatory functions, we compared the relative quantities of the immune-stimulatory miRNA species with the snRNA species U6, as this RNA-species is not associated with immune-regulatory effects. Consistent with this idea, U6 was slightly elevated in EV derived from DC incubated with non-activated bystander T-cells, but not in EV from DC incubated with activated bystander T-cells. Collectively, these findings suggest that incorporation of immune stimulatory miRNA species, such as miR146a and miR155, into EV derived from LPS-experienced moDC is selectively upregulated in response to activated bystander T-cells.

**Figure 4:**
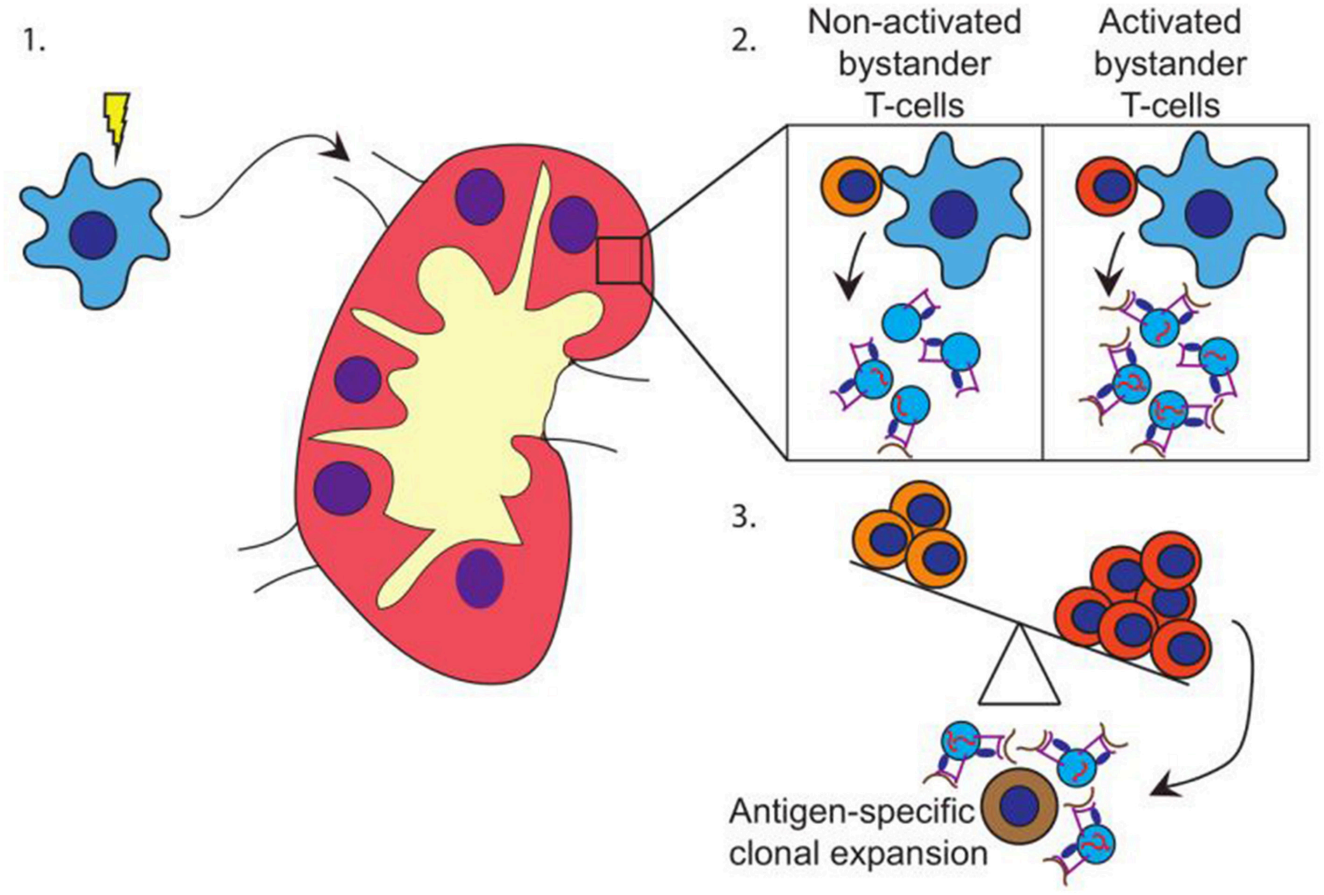
Hypothesis. **(1)** DC (blue) encounter maturation stimuli and migrate toward the (draining) lymph node. **(2)** In the lymph node, DC encounter both activated and non-activated bystander T-cells. Upon the interaction with activated bystander T-cells, DC release EV containing HLA class I-pathogen-derived peptide complexes and immune-stimulatory RNA-species. **(3)** Depending on the ratio of activated vs. non-activated bystander T-cells, the DC releases EV with a capacity to activate primed cognate T-cells. EV may also stimulate T-cells via cross dressing of bystander DC (not shown).

Based on our data, we propose a model in which pathogen-experienced DC travel toward the lymph node (Figure 4-1) to encounter both activated and non-activated bystander T-cells, the ratio of which is dependent on the prevailing immune-status in that specific lymph node. Interactions with activated bystander T-cells further stimulate the activation status of the DC, and induce the release of EV by the activated DC in the lymph node. These EV act are enriched for molecules that can stimulate antigen specific T-cells, including antigen presenting HLA-I loaded with pathogen-derived peptides, as well as co-stimulatory molecules, membrane organizing proteins, and immune-modulatory RNA-species (Figure 4-2). The EV may stimulate cognate T-cells directly, or via bystander DC, ultimately resulting in cognate T-cell activation. Thus, the pre-existing balance of activated and non-activated bystander cells in the lymph node helps shaping the immune response that is generated against the pathogen presented by the DC (Figure 4-3).

The promise of DC-derived EV as cancer-therapy was posed in the early stages of EV-research (18). Since then, patient DC-derived EV have been tested in clinical trials for their capacity to activate T-cell responses toward clearing cancer cells, but only limited success has been reported until now (41–43). One potential complication is that DC-derived EV can have tolerogenic or stimulatory capacities, and it is ill-defined which experimental conditions drive the generation of such phenotypically opposing EV. Our data, obtained with human cells, show that autologous activated bystander T-cells induce the release of DC-derived EV and endow them with immune-stimulatory capacities. These observations may help further optimization of DC-derived EV-based vaccines.

## Data Availability

The raw data supporting the conclusions of this manuscript will be made available by the authors, without undue reservation, to any qualified researcher.

## Author Contributions

ML designed and performed experiments, analyzed data, and wrote the manuscript. DK, EB, EvV, and TD performed experiments. RW supervised the design and execution of microscopy experiments. WS and MB oversaw the project, helped designing experiments, and wrote the manuscript.

### Conflict of Interest Statement

The authors declare that the research was conducted in the absence of any commercial or financial relationships that could be construed as a potential conflict of interest.
