# Supplementary figures and images for: Bystander T-Cells Support Clonal T-Cell Activation by Controlling the Release of Dendritic Cell-Derived Immune-Stimulatory Extracellular Vesicles
Source: Front Immunol. 2019 Mar 12;10:448. doi: 10.3389/fimmu.2019.00448 (PMC6423080; doi:10.3389/fimmu.2019.00448)

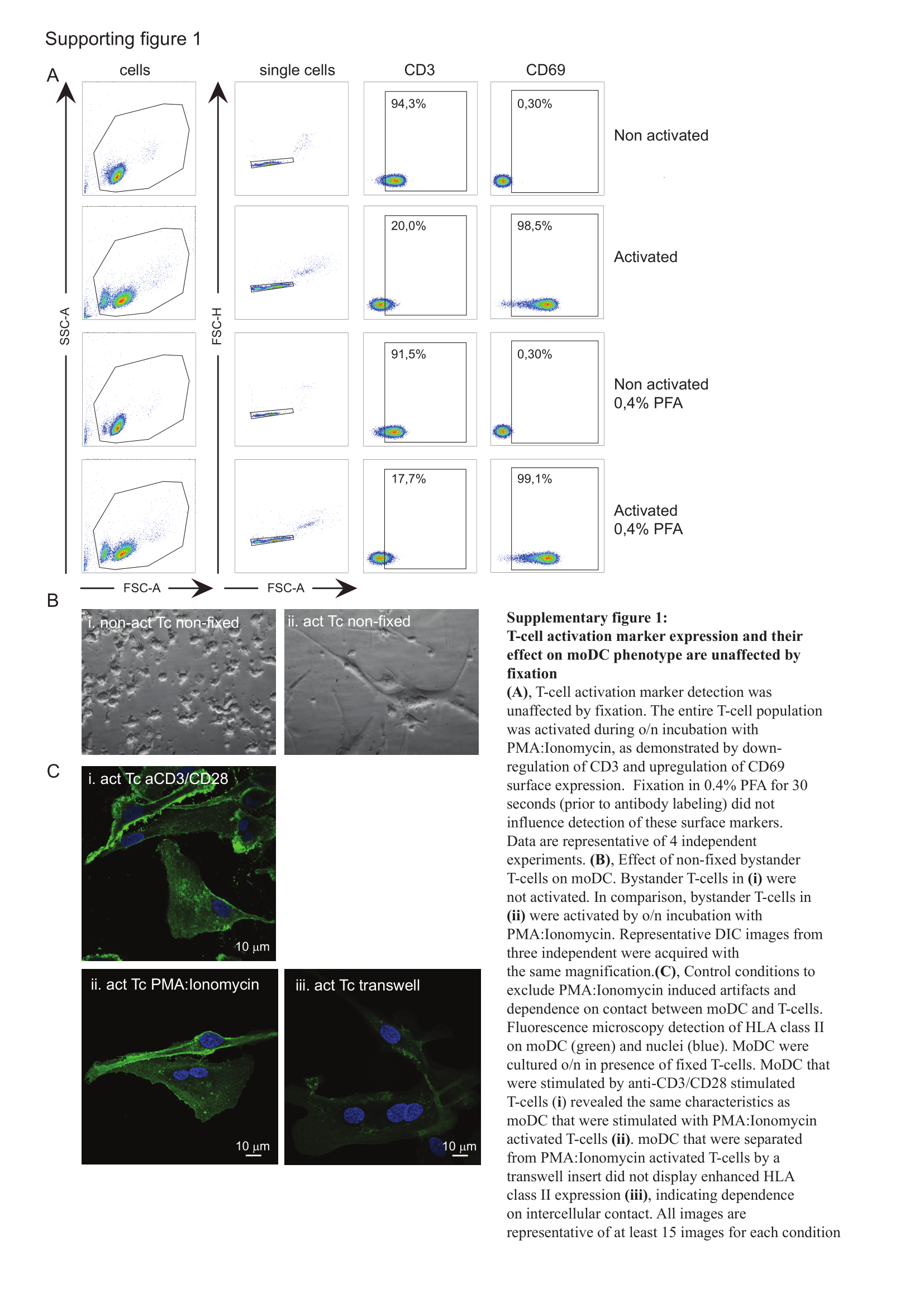

Supplement: Supplementary file 1 [file Image_1.tiff]

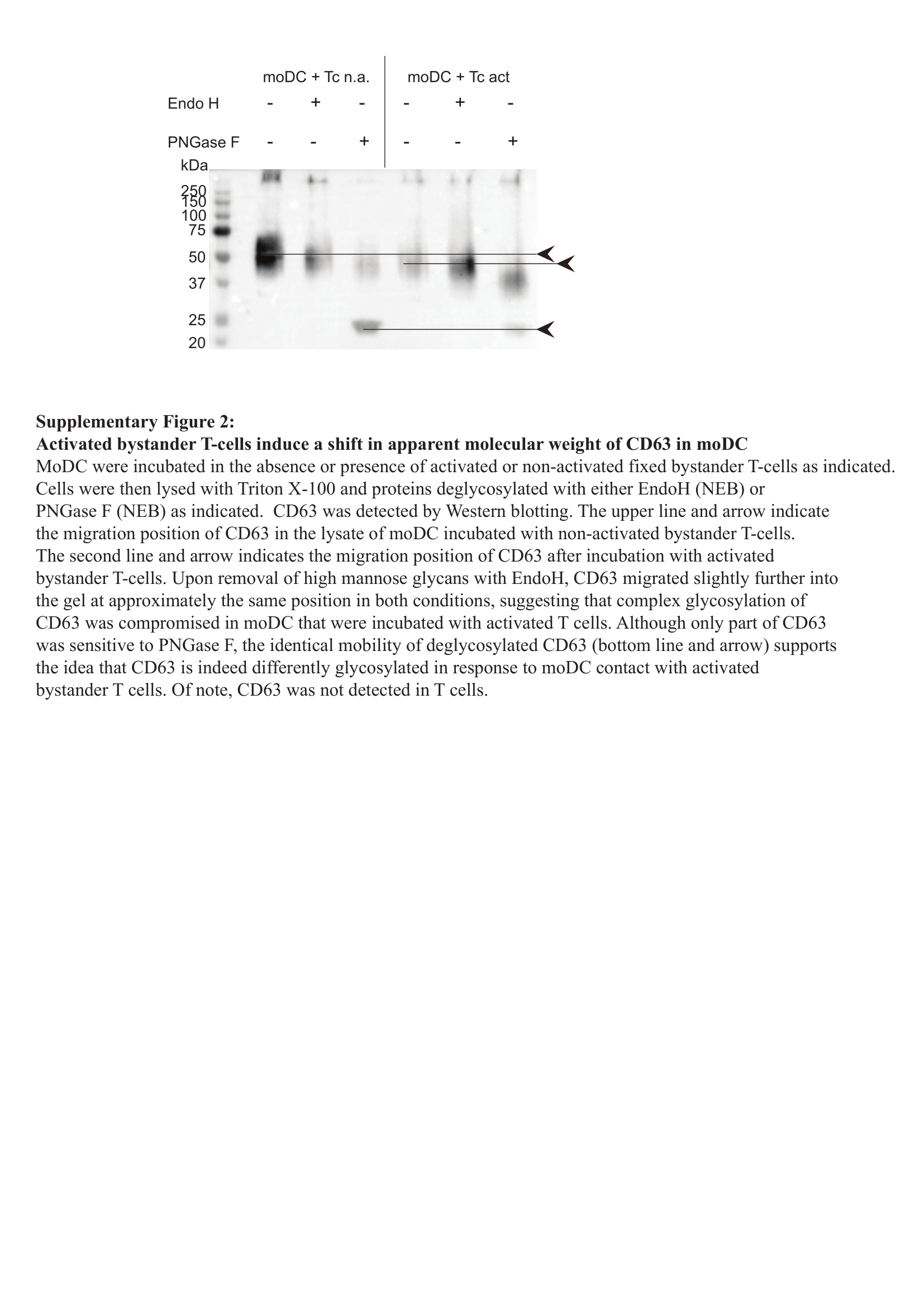

Supplement: Supplementary file 2 [file Image_2.tiff]
